# Distinct Effects of Abelson Kinase Mutations on Myocytes and Neurons in Dissociated *Drosophila* Embryonic Cultures: Mimicking of High Temperature

**DOI:** 10.1371/journal.pone.0086438

**Published:** 2014-01-21

**Authors:** Lijuan Liu, Chun-Fang Wu

**Affiliations:** 1 Key Laboratory of Developmental Genes and Human Disease, Institute of Life Sciences, Southeast University, Nanjing, China; 2 Department of Biology, University of Iowa, Iowa City, Iowa, United States of America; University of Vienna, Max F. Perutz Laboratories, Austria

## Abstract

Abelson tyrosine kinase (Abl) is known to regulate axon guidance, muscle development, and cell-cell interaction *in vivo.* The *Drosophila* primary culture system offers advantages in exploring the cellular mechanisms mediated by Abl with utilizing various experimental manipulations. Here we demonstrate that single-embryo cultures exhibit stage-dependent characteristics of cellular differentiation and developmental progression in neurons and myocytes, as well as nerve-muscle contacts. In particular, muscle development critically depends on the stage of dissociated embryos. In wild-type (WT) cultures derived from embryos before stage 12, muscle cells remained within cell clusters and were rarely detected. Interestingly, abundant myocytes were spotted in *Abl* mutant cultures, exhibiting enhanced myocyte movement and fusion, as well as neuron-muscle contacts even in cultures dissociated from younger, stage 10 embryos. Notably, *Abl* myocytes frequently displayed well-expanded lamellipodia. Conversely, *Abl* neurons were characterized with fewer large veil-like lamellipodia, but instead had increased numbers of filopodia and darker nodes along neurites. These distinct phenotypes were equally evident in both homo- and hetero-zygous cultures (*Abl/Abl* vs. *Abl*/+) of different alleles (*Abl^1^ and Abl^4^*) indicating dominant mutational effects. Strikingly, in WT cultures derived from stage 10 embryos, high temperature (HT) incubation promoted muscle migration and fusion, partially mimicking the advanced muscle development typical of *Abl* cultures. However, HT enhanced neuronal growth with increased numbers of enlarged lamellipodia, distinct from the characteristic *Abl* neuronal morphology. Intriguingly, HT incubation also promoted *Abl* lamellipodia expansion, with a much greater effect on nerve cells than muscle. Our results suggest that Abl is an essential regulator for myocyte and neuron development and that high-temperature incubation partially mimics the faster muscle development typical of *Abl* cultures. Despite the extensive alterations by *Abl* mutations, we observed myocyte fusion events and nerve-muscle contact formation between WT and *Abl* cells in mixed WT and *Abl* cultures derived from labeled embryos.

## Introduction

The mammalian cytoplasmic Abelson tyrosine kinase gene (*c-abl*) is an oncogene first revealed by a specific chromosomal translocation, the Philadelphia chromosome [1], found in patients with chronic myeloid leukemia [2], [3]. Studies in vertebrate species have established that Abelson kinase is also important for other cell types, primarily through cytoskeleton organization through actin dynamics regulation [4], [5].

In *Drosophila*, the homologous gene *Abl* (initially described as *abl)* [6] is known to exert wide-ranging effects in development and growth. For example, *Abl* mutant embryos display arrested motor axon outgrowth when targeting peripheral muscles [7]. Furthermore, interactions with *fasciclin 1* (*fas1*), which encodes an adhesion molecule, further disrupts growth cone guidance and nerve track formation in the embryonic central nervous system (CNS) in *Abl fas I* double mutants [8]. Abl is shown to regulate growth cone motility mediated by actin cytoskeletal organization that is tightly regulated by its phosphorylation substrate Ena (homolog of VASP (Vasodilator-Stimulated Phosphoprotein) in mammals) [9], [10]. While less extensively studied in myocytes, *Abl* has been shown to interact with the gene *disabled* (*dab*), causing musculature derangement in double-mutant embryos [11].

In the present study, we furthered the above *in vivo* findings by utilizing embryonic cell culture system. Our previously work has utilized neuronal cultures derived from dissociated larval CNS [12], [13], [14], [15], [16] or embryonic “giant” neurons cultures from cell division-arrested neuroblasts, in which Cytochalasin B treatment eliminates muscle cells [17], [18], [19], [20]. To extend our observations to other cell types, in addition to neurons, we carried out experiments using the single-embryo culture system to study muscle cell development and nerve-muscle interaction.

The dissociated cultures were initiated at defined embryonic stages, which enabled us to study the developmental progression of distinct cell types and the interactions among them, as well as to distinguish between mechanisms mediated by cell-cell interactions or cell autonomous processes.

Here we report several findings that have not been characterized previously. First, muscle development in our cultures critically depended upon the stage at which embryos were dissociated. Second, *Abl* mutations differentially affect various aspects of myocyte and neuronal development. In particular, abundant muscle cells were present in *Abl* cultures dissociated at embryonic stage 10, while muscle cells were not seen in WT cultures until stage 12. Third, high temperature (HT, 30°C) incubation greatly enhanced neuronal and muscle growth and partially mimicked *Abl* myocyte phenotypes. Fourth, *Abl* nerve and muscle cells responded differentially to HT incubation, supporting the notion of distinct interacting partners of *Abl* in nerve and muscle development.

## Materials and Methods

### Drosophila Stocks

The primary wild-type (WT) strain was Canton S (CS), which was used for all statistics and images, except for Rhodamine 123 staining, where a second WT strain, Oregon-R (OR), was used. Two *Abl* alleles, *Abl^1^* and *Abl^4^*, were kept in marked and balanced stocks: *Abl^1^ kar red e/TM6B, Tb* (from Bloomington Stock Center, Bloomington, IN) and *Abl^4^ kar red e/TM6B, Tb* (from Dr.FM Hoffmann), and were used for making the appropriate crosses as specified in our culture studies. The balancer line w*; *Sb^1^/TM3, P {ActGFP} JMR2, Ser^1^* (Bloomington Stock Center, Bloomington, IN) was used to replace unlabeled balancer TM6B to make labeled heterozygous *Abl* alleles to facilitate genetic background checks in culture. All the *Abl* alleles used for cultures are *Abl^4^/TM3-ser-GFP* and *Abl^1^//TM3-Ser-GFP*. CS was crossed with both *Abl^4^/TM3-ser-GFP* and *Abl^1^//TM3-ser-GFP* to yield heterozygous *Abl* alleles under wild-type background for cultures. The ubiquitously expressed driver, *daughterless* (Da-Gal4) (Gift from Toshi Kitamoto) and the pan-neuronal driver, Elav-Gal4, were used to drive UAS-GFP, UAS-RFP (Bloomington Stock Center, Bloomington, IN).

### Embryonic Primary Culture

The methodology for preparing single-embryonic cultures is a modified version from two previously described protocols for the *Drosophila* larval central neuron system (CNS) culture [12], *Drosophila* embryonic “giant” neuron culture system [17] and primary neuron cultures of *Drosophila* [21]. The culture system in this paper essentially is based on the “giant” *Drosophila* embryonic culture system without adding cytochalasin B into medium. Briefly, the stage 7–8 embryos were selected by the clear gastrulation marker, after egg laying 3∶00 h–3∶40 h; the stage 7–8 embryos were incubated in medium for an additional 2 hours, where the embryos progressed to late stage 10; 4 hrs after stage 7–8, embryos progressed to early stage 12; 7 hrs after stage 7–8, embryos progressed to stage 13. Tissues of the embryos were sucked by micropipette, and immediately plated in culture medium on a coverslip. The coverslips were put into moisture chambers, which were in turn placed in either room temperature (22–25°C) or a 30°C incubator for heat stress treatment. Throughout the study, observations on long-term HT effects were achieved by placing the moisture chambers in an incubator set to 30°C. The cultures were exposed to HT throughout the growth period, up until the time of microscope observation, as specified. The culture method for Figure S2 was based on previous research [22], [23]. We homogenized 4–6 embryos from stage 10 by eppendorf micropestle in 80 µl culture medium, pelleted in a microcentrifuge at 5,000 rpm for 5 minutes at room temperature, resuspended by pipetman, and plated directly.

The culture medium contains 80% *Drosophila* Schneider medium and 20% fetal bovine serum (GIBCO, Invitrogen, Carlsbad, CA) with the addition of 200 ng/ml insulin, 50 g/ml streptomycin, and 50 U/ml penicillin (all from Sigma, St. Louis, MO). We carried out single-embryo and two-embryos mixed cultures. For *Abl^4^/TM3-ser-GFP* and *Abl^1^//TM3-ser-GFP* cultures, we checked maternal fluorescent signal after plating 2 days. Cultures of *Abl* alleles used for analysis were from both hetero and homozygous stocks, both of which showed similar phenotypes.

### Fluorescent Staining

#### Immunohistochemical staining

The staining protocol was based on our previous protocol for larval CNS culture [24]. Cultures were briefly washed in phosphate-buffered saline (PBS, pH 7.2), fixed for 10 minutes with 3.7% formaldehyde, washed in PBS 5 times for 5 minutes each, and permeabilized with 0.01% Triton X-100 for 2 minutes. After blocking with 3% bovine serum albumin (BSA) for 10 min, cells were washed 5 times for 5 minutes each, incubated at room temperature for 25 minutes with TRITC-conjugated phalloidin (0.05 mg/ml, Sigma), followed by 5 washes in PBS. After staining, they were mounted in VECTASHIELD® Mounting Medium with DAPI to stain nuclei or without DAPI (4′, 6-diamidino-2-phenylindole) (Vector Lab). The cultures were observed and photographed on a Leica inverted microscope.

#### Rhodamine 123 staining

Cultures were incubated with 1 µg/ml Rhodamine 123 (molecular probes) in *Drosophila* culture medium for 2 minutes, washed with fresh medium 3 times, and then mounted in culture medium immediately for observation.

### Image Processing and Quantification

We used 20X objective lens (N.A. = 0.4, Nikon) on an inverted microscope (Nikon, Japan), 40X objective lens (N.A. = 0.55,Leica) and 100X oil immersion objective lens (N.A. = 1.3,Leica) on an inverted microscope (DM IRBE, Leica, Wetzlar, Germany) or an upright microscope (DM RBE, Leica, Wetzlar, Germany) for collecting images of cultured cells. Images were processed and analyzed using ImageJ, Adobe Photoshop CS, Gimp and Inkscape. ImageJ was used for quantifications of filopodia length, and lamellipodia area. The cells clusters counted were less than 30 cells with an area of cell bodies occupying less than 2500 µm^2^. To be counted, filopodia along neurites had to be at least 5 µm away from cell bodies. Muscle lamellipodia were defined as muscle terminals with a flattened, well-expanded area of at least 5 µm^2^.

## Results

### Neurons and Muscle Cells Grown in Dissociated Cultures: Embryonic Stage-dependent Myocyte Development

In this study, we performed single-embryo cultures grown on uncoated glass coverslips to observe the growth and differentiation potentials of dissociated nerve and muscle cells, as well as their interactions. Individual embryos collected at different times after egg-laying were staged and prepared for plating under a dissecting microscope. We strove for the optimal extraction of entire contents from individual embryos with a micropipette. The tissue was subsequently extruded and dispersed in a drop of medium on the coverslip surface, without mechanical agitation or enzyme treatment. Consequently, both isolated cells and cell clusters were present in the culture. Presumably, cell groups with stronger adhesion were more likely to remain in associated cell aggregates.

**Figure 1:**
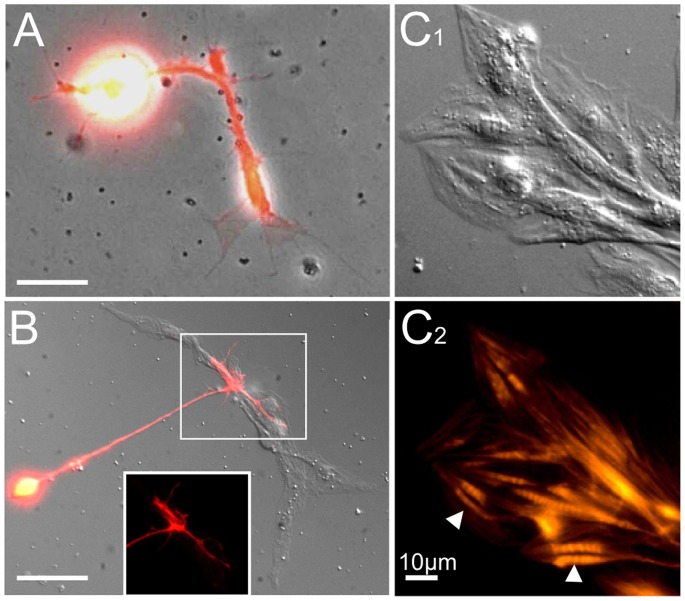
Neurons, muscle cells and nerve-muscle interaction observed in single-embryo cultures dissociated from different embryonic stages. A) A merged image of phase-contrast and fluorescent optics. Elav-Gal4 and UAS-RFP were used to produce the red fluorescent signal in stage 10 neurons (100X). Note the typical growth cone with expanded lamellipodia and filopodia. B) Merged image of cell interactions between a neuron labeled with Elav-Gal4 driven UAS-RFP and muscle cells from late stage 12 (DIC, 100X). The neuron has established extensive contact with a group of muscle cells. Inset: red fluorescent signal from neurite terminals over the muscle group. (A–B) Cultures were incubated 4 days. C) A well-differentiated group of muscle cells (40X) from stage 13 grown in culture for 7 days observed with DIC (C1) and phallodin fluorescent (C2) optics. Note the flattened lamellipodia and multiple nuclei, as well as muscle striation heighted by phalloidin staining (arrowheads), signifying maturity of the muscle cells. Scale bars, 10 µm.

**Figure 2:**
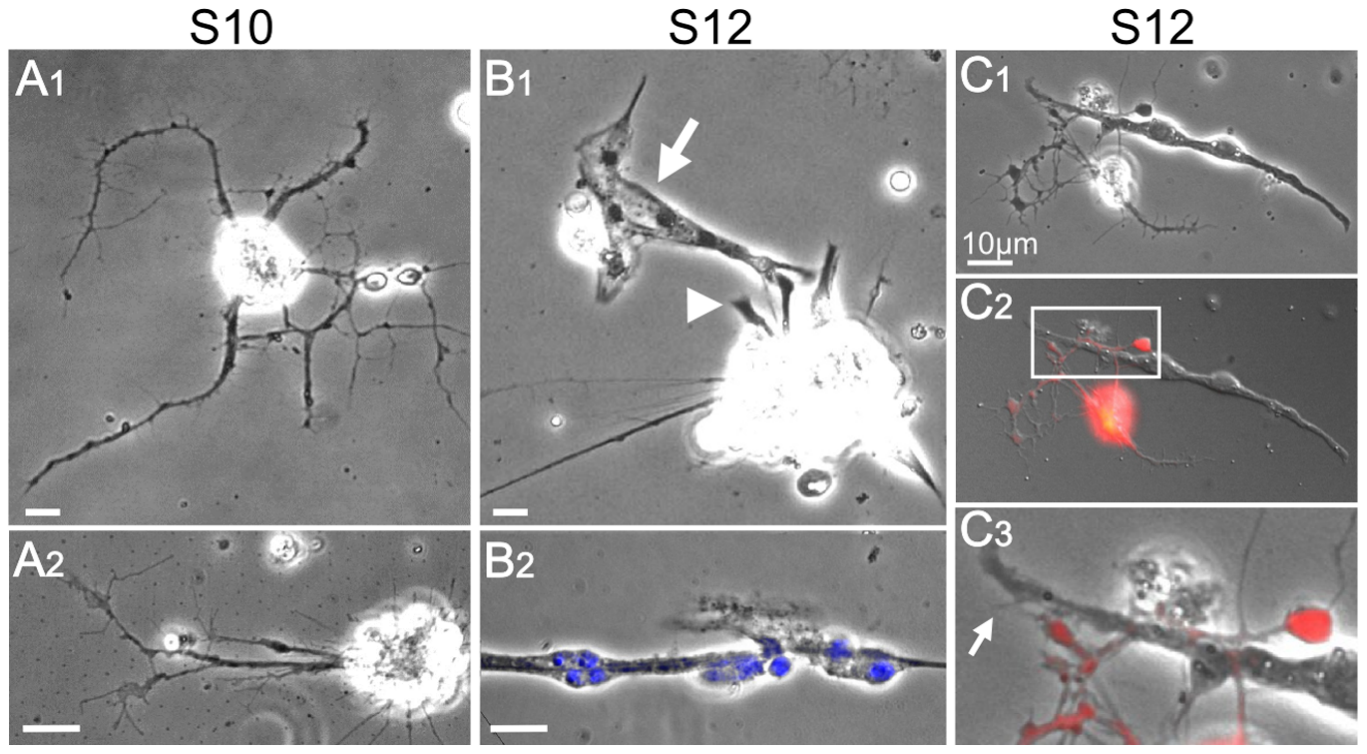
Migration and fusion of muscle cells in dissociated cultures depends on the embryo stage. A–B) Phase contrast images of typical cell groups from stage 10 and stage 12. A1) Stage 10 neuron cells (40X). A2) Stage 10 neuron cells (100X). B1) Stage 12 muscle cells isolated (arrow) and involved in cell clusters (arrowhead). B2) Stage 12 multinucleated, fused muscle cells. Phase image merged with DAPI staining (100X). C) Interaction between developed muscle and nerve cells from a stage 12 embryo (100X). C1) Phase contrast. C2) DIC image merged with red fluorescent signal from Elav-Gal4 driven UAS-RFP to identify neurons. C3) Enlargement of the boxed area in C2 displayed in phase contrast optics. The arrow indicates an example of areas of interaction between neuronal filopodia (labeled in red) and muscle microspikes. All cultures were in incubated 2–5 days. Scale bars, 10 µm.

In such cultures, both nerve and muscle cells could be successfully grown, but the appearance of myocytes critically depended upon the developmental stage at which the embryonic tissue was extracted and plated. As Figure 1 shows, there were abundant muscle cells in cultures dissociated from older embryos (stage 13 and beyond) that displayed differentiated features, e.g. striations and occasional contractions, when grown at room temperature for more than 1 day (Fig. 1C; data not shown). Generally, in cultures from stage 12 or older embryos, muscle cells could be readily found in open fields of coverslip a few hours after plating, whereas stage 10 cultures rarely produced any isolated muscle cells, even after several days of incubation, while neuronal development proceeded vigorously with extended neurites decorated with filopodia and lamellipodia (Fig. 2). Thus, there was an apparent critical transition between embryonic stage 10 and stage 12 for myocyte development in single-embryo cultures.

It should be noted that cultures prepared from stage 10 embryos occasionally gave rise to a few myocytes in open fields (data not shown). However, further incubation over several days indicated poor survival of these myocytes and no additional myocytes appeared in culture (Fig. 2A). Conversely, in stage 12 cultures, myocyte aggregates of various group sizes continued to grow and differentiate into multinucleated fused cells beyond 4 days and up to 18 days (Fig. 2B). Additionally, neuromuscular contacts were frequently encountered in these cultures (Fig. 2C).

Notably, in stage 12 cultures, there were two distinct muscle locations; either isolated from neuronal clusters (Fig. 2B1, arrow) or still in the process of emerging from nerve tissue (Fig. 2B1, arrowhead). In both cases, myotube differentiation continued as indicated by the fused, multinucleated muscle cells (Fig. 2C) that often exhibited contraction behavior with or without innervation. Time-lapse observations confirm myocyte movements and changes in shape during this translocation process (data not shown).

This result suggests that in our single-embryo cultures, the tendency of myocyte migration from tissue aggregates and the abundance of individual or groups of myocytes in open fields increases with embryo age. This apparently reflects the *in vivo* observations that stage 10 embryos contain undifferentiated myoblasts and muscle cell proliferation does not occur until stage 12 [25].

### Abelson Kinase Mutations Exert Dominant Effects on Myocyte Development and Neuronal Growth in Culture

In our previous study, we found altered neuronal morphology and growth in dissociated larval CNS cultures of *Abl* mutants, in which defective cytosolic tyrosine kinase Abl leads to increased plating efficiency, enhanced growth and motility of filopodia, and altered neurites with swollen nodes and growth cone morphology [24]. These cellular phenotypes are likely derived from disrupted actin cytoskeleton regulation.

**Figure 3:**
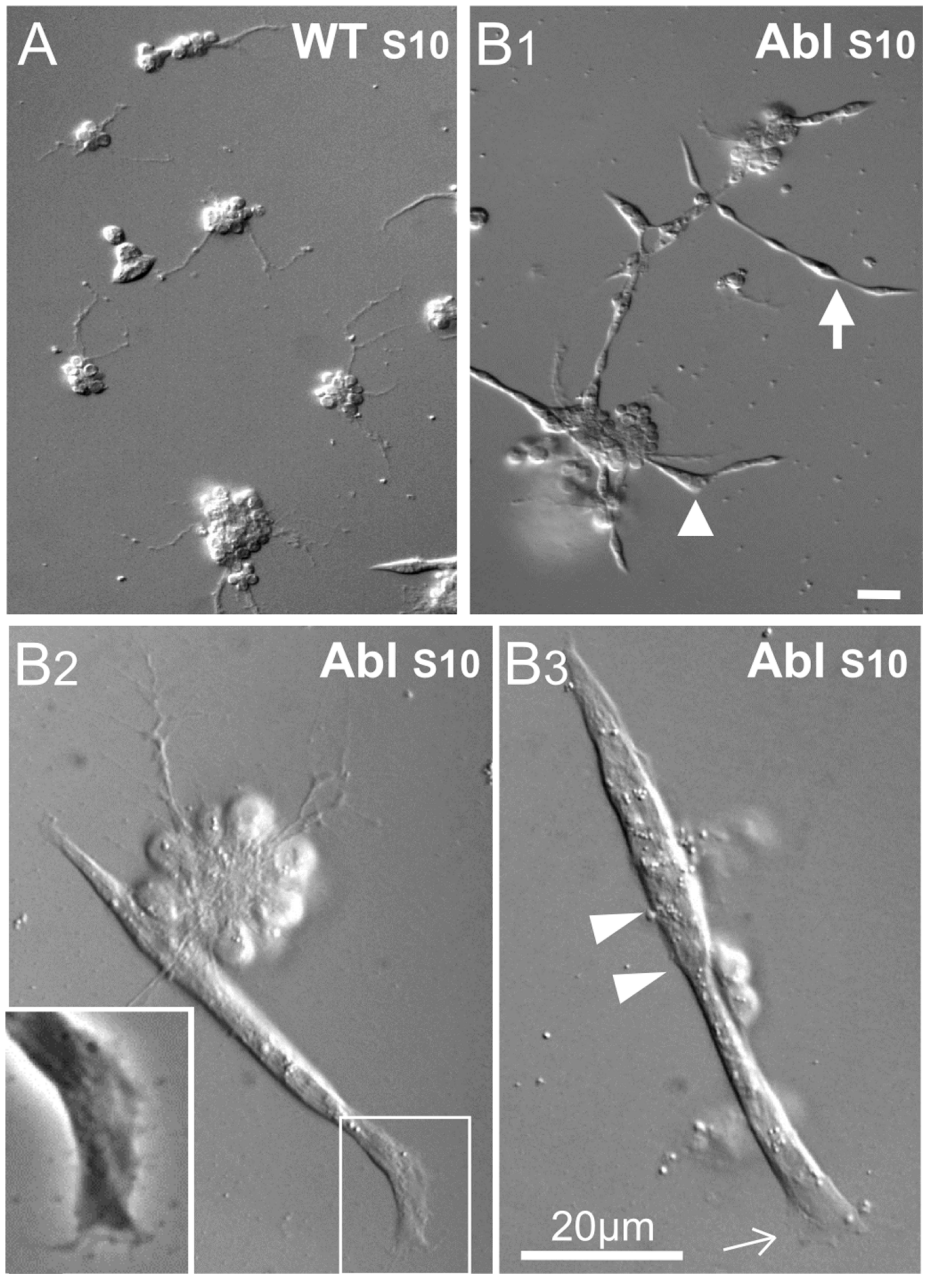
Cultures dissociated from earlier stage of *Abl* embryos display muscle cell movement and fusion. Sample DIC images of room temperature cultures derived from stage 10 WT and *Abl* embryos. (A & B1) WT cultures rarely display muscle cells, whereas *Abl* cultures clearly show developing muscle cells (40X), both in isolation (arrow) and in association with cell clusters (arrowhead). Cultures were incubated 37 h. B2) A fused *Abl* muscle cell associated with a neuron cluster (100X). The phase contrast inset of boxed region shows flattened and extended lamellipodia. B3) An isolated *Abl* muscle cell (100X). Note the lamellipodia (thin arrow) and nuclei (arrowheads). (B2&B3) cultures were incubated 2 days. Scale bars, 20 µm.

In the present single-embryo culture system, we discovered striking effects of Abl on embryonic muscle development in addition to abnormal neuronal growth. We observed abundant isolated muscle cells in open fields of coverslips as well as muscle cells emerging from, but still associated with, neuronal clusters in cultures prepared from embryos as early as stage 10 (Fig. 3).

**Figure 4:**
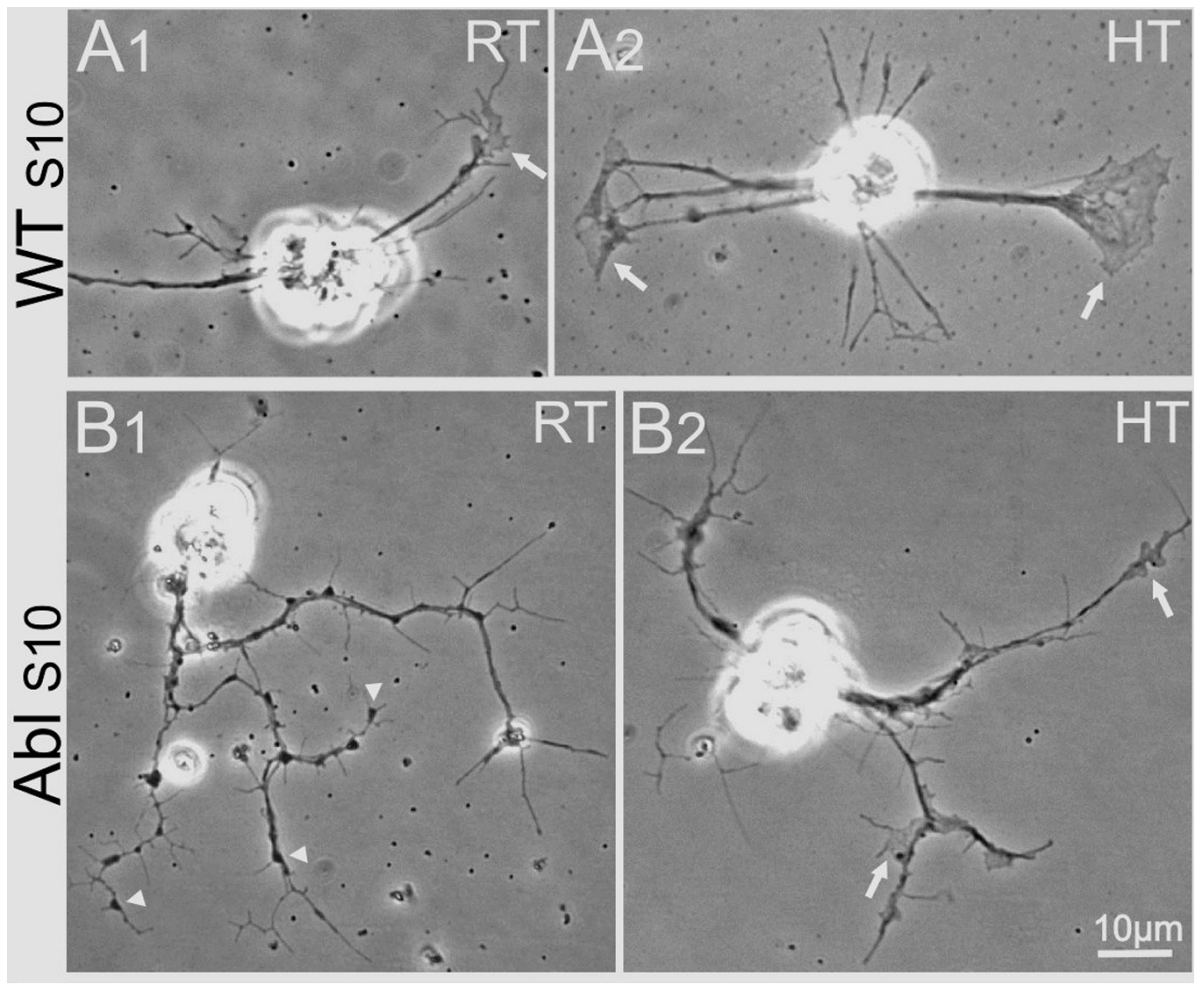
Comparison of the Abelson Kinase mutation and high-temperature effects on neuronal growth. Phase contrast images (100X) of stage 10 WT and *Abl* cultures grown at room temperature (RT) or high temperature (HT, 30°C). A1 vs. B1) In RT cultures, *Abl* neurons show enhanced growth of filopodia compare to WT. There is also an increase in dark nodules along the neurite and at the terminal (B1, arrowheads). However, while phase-light growth cones with expanded lamellipodia are rare in *Abl*, they can readily be seen in WT (A1, arrow). A1 vs. A2) HT incubation of WT neurons produces extremely large growth cones (A2, arrows). B1 vs. B2) HT incubation of *Abl* neurons enhances development of phase-light lamellipodia at growth cones and along neurites (B2, arrows). All cultures were incubated 3–6 days. Scale bars, 10 µm.

**Figure 5:**
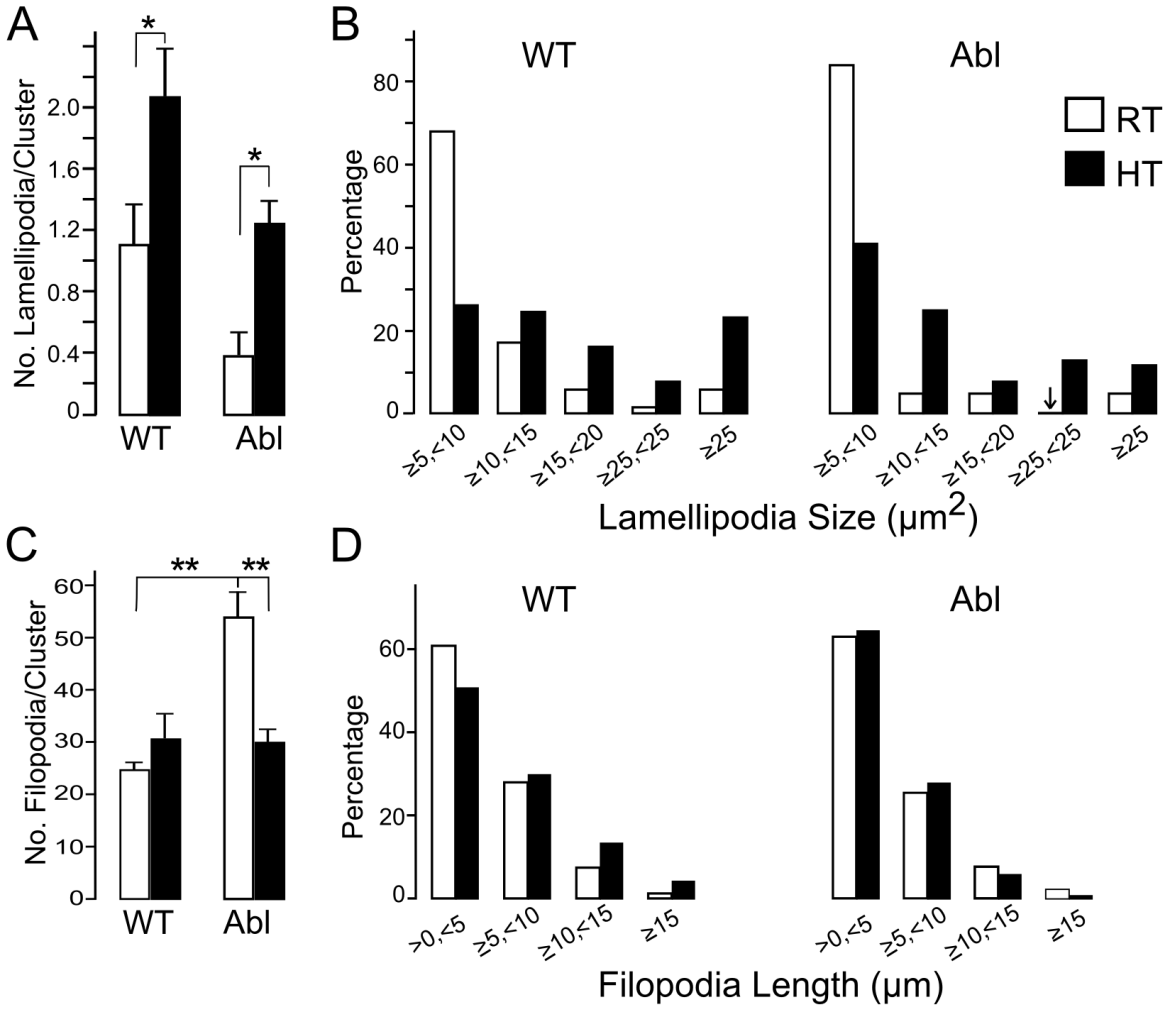
Analysis of WT and *Abl* neuronal growth at RT and HT. Numbers of lamellipodia per cell cluster in WT and Abl cultures incubated at HT and RT. (Only lamellipodia with area >4 µm2 are included.) HT incubation of WT produced a significant increase in lamellipodia in both WT and Abl cultures. Cell clusters numbers: WT RT, 107; WT HT, 165; Abl RT,92; Abl HT, 113, respectively, *p<0.05, unpaired T-test. B) Distribution of lamellipodia area (µm2). Lamellipodia are enlarged significantly at HT in both WT and Abl cultures (WT, p<0.0001; Abl, p<0.0001; χ2 test). Lamellipodia numbers: WT RT, 133; WT HT, 175; Abl RT, 19; Abl HT, 151, respectively. Note that over 60% of WT lamellipodia at RT fall between 5–10 µm2, whereas at HT over 70% of lamellipodia are 10 µm2 or larger, even reaching areas over 25 µm2. In contrast, 80% of lamellipodia at RT are less than 10 µm2, whereas at HT over 60% are 10 µm2 or larger in Abl cultures. C) Numbers of filopodia per cell cluster. Neurons in Abl cultures show a greater number of filopodia at RT than WT but show a significant decrease in filopodial numbers following HT incubation (cell clusters numbers: WT RT, 74; WT HT, 66; Abl RT, 72; Abl HT, 102, respectively, **p<0.01, unpaired T-test). D) Distribution of filopodial lengths (µm). HT incubation does not produce a significant effect on filopodial length. There is a slight tendency of increased abundance of longer filopodia in WT cultures following HT incubation but χ2 test shows no significant differences. For each culture conditions at least 3 coverslips were used and sequential Bonferroni adjustments for multiple comparisons were performed. All cultures were derived from stage 10 embryos and incubated 2–5 days. Error bars, SEM. The statistics here and in Figure 7 are based on pooled data from *Abl4/+, Abl1/+*, *Abl4/Abl4*, and *Abl1/Abl1*. See text.

While WT cultures derived from embryonic stage 10 were devoid of exposed, readily detectable muscle cells (Fig. 3A), *Abl* cultures were populated with single or multinucleated muscle cells, both isolated and still connected with cell clusters (Fig. 3B1). These *Abl* muscle cells, including multinucleated, fused myocytes, displayed well-expanded lamellipodia with filopodia projections (Fig. 3B2–B3 & see HT effects below). Consistent with the altered neuronal growth in the larval CNS culture [24], neurons in *Abl* cultures displayed increased numbers of filopodia and more swollen, dark nodes along neurites (Fig. 4B1). However, compared to WT control, *Abl* neurons exhibited tendency to show fewer veil-like structures and well developed, large lamellipodia (>4 µm^2^) occurred only at a lower frequency (Fig. 5).

As stated in Methods, the majority of homozygous *Abl* mutant alleles are lethal at pupal or earlier stages, so we performed most of the experiments on *Abl^4^/+* (about 70% of total cultures) and some on *Abl^1^/+* (about 10%) heterozygous embryos, both of which demonstrated striking phenotypes that were readily detected. We have also collected data from *Abl^1^/Abl^1^* embryos (about 10%) as well as some *Abl^4^/Abl^4^* embryos (less than 10%). Surprisingly, the neuronal and myocyte phenotypes observed were similar in all four types of culture (Figure S1). The representative photomicrographs presented in this paper were collected from *Abl^4^* allele cultures, but the statistics for phenotypic characterization shown in the bar charts throughout this report are based on pooled data from cultures of all four genotypes. Furthermore, we observed a few cultures derived from *Abl^1^/Abl^4^* embryos and found similarly extreme phenotypes of neurons and myocytes (data not shown). The above results indicate that both *Abl^4^* and *Abl^1^* mutations exhibit dominant effects on both nerve and muscle cell development.

### Enhanced Neuronal Growth Promoted by High-temperature Incubation in WT and *Abl* Cultures

The above observations suggest that cytosolic Abl tyrosine kinase may play differential roles in regulating veil-like structures, such as growth cone lamellipodia and muscle lamellipodia, in nerve and muscle development. To accentuate these potential differences, we subjected these two cell types to temperature stress since our previous research has shown striking high temperature (HT)-induced increases in growth cone size and terminal arborization in embryonic “Giant” neuron cultures [26].

In stage 10 WT cultures, we found that incubation at HT (30°C) tended to increase the number of neuronal lamellipodia over room temperature (RT) WT cultures (Fig. 5A). Strikingly, HT incubation of stage 10 *Abl* produced a drastic increase over RT in the number of neuronal lamellipodia (>300%), raising the average to levels comparable to those found in WT (Fig. 5A). Furthermore, HT-incubated neurons in both WT and *Abl* displayed enhanced neuronal growth via larger lamellipodia with greatly enlarged sizes (>10 µm^2^), which are normally less than 10 µm^2^ at RT (Fig. 5B). Therefore, HT incubation clearly enhanced neuronal growth through the increase in lamellipodia size. Additionally, the length of filopodia projecting along neurites tended to be increased in HT-incubated WT (Fig. 5D). There were more lengthened (>10 µm long) filopodia at HT, which were rare in cultures grown at RT.

However, the number of filopodial projections along neurites decreased in *Abl* cultures (Fig. 5C–D), further demonstrating that Abl regulates lamellipodia and filopodia in different ways.

### High-temperature Incubation of WT Cultures Mimics *Abl* Muscle Phenotypes but Exerts Little Effect on Cultured *Abl* Myocytes

**Figure 6:**
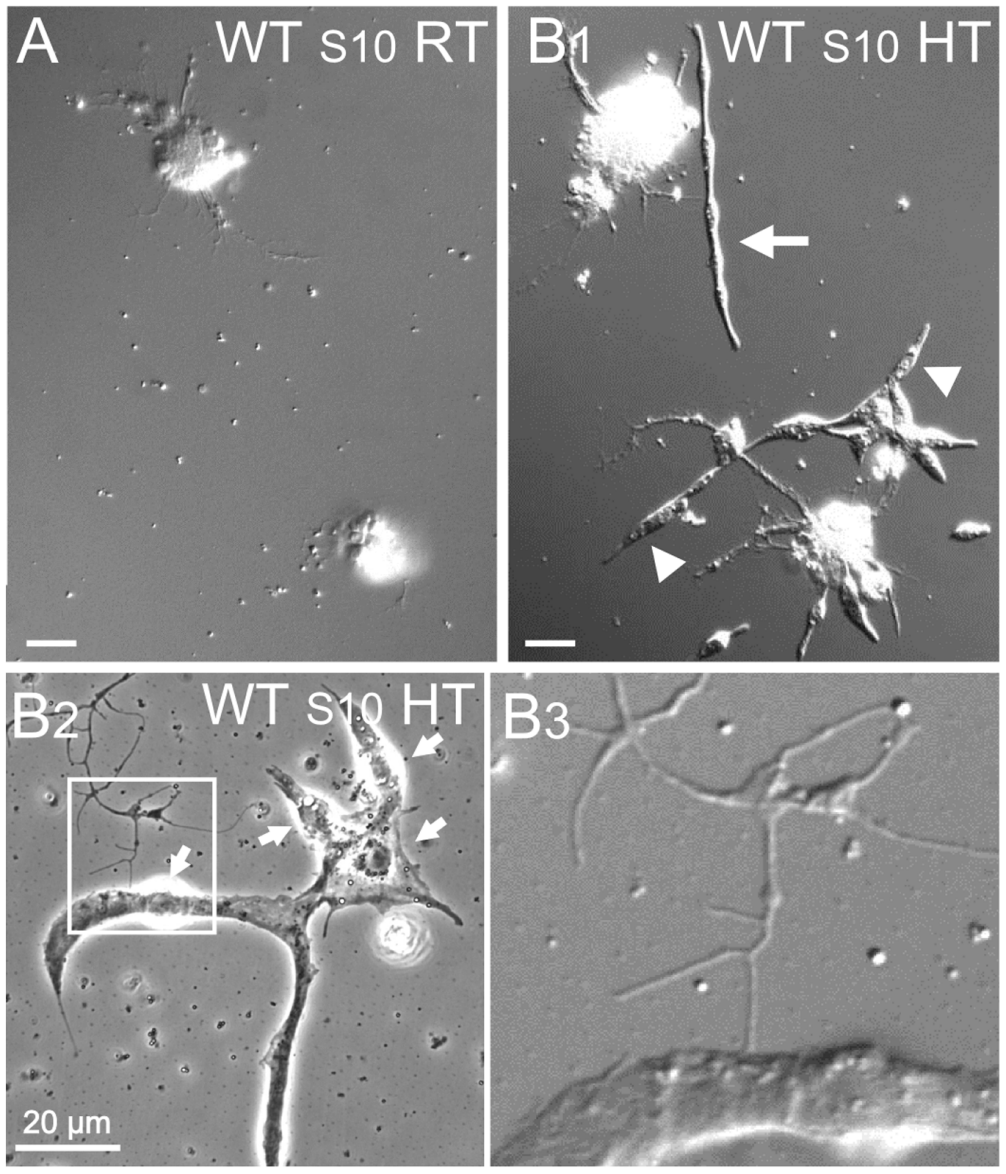
High-temperature incubation promotes muscle development in cultures from stage 10 WT embryos. A) WT cultures from stage 10 embryos rarely presented any muscle cells when incubated at RT (40X). B1) HT incubation of stage 10 WT cultures produced abundant muscle cells both in isolation and in association with cell clusters. Sample from culture after 39 h incubation at HT (40X), showing a muscle cell isolated from cell aggregations (arrow) and still others associated with cell clusters (arrowheads). B2–B3) WT cultures after 9 days incubation at HT (100X). B2) An unusually large WT multinucleated muscle cell (arrows pointing to nuclei) interacting with neurites from a neuron (soma not shown). B3) Enlarged image of boxed area in B2 displaying a neuromuscular contact. B2, phase contrast; A, B1, and B3, DIC images. Scale bars, 20 µm.

**Figure 7:**
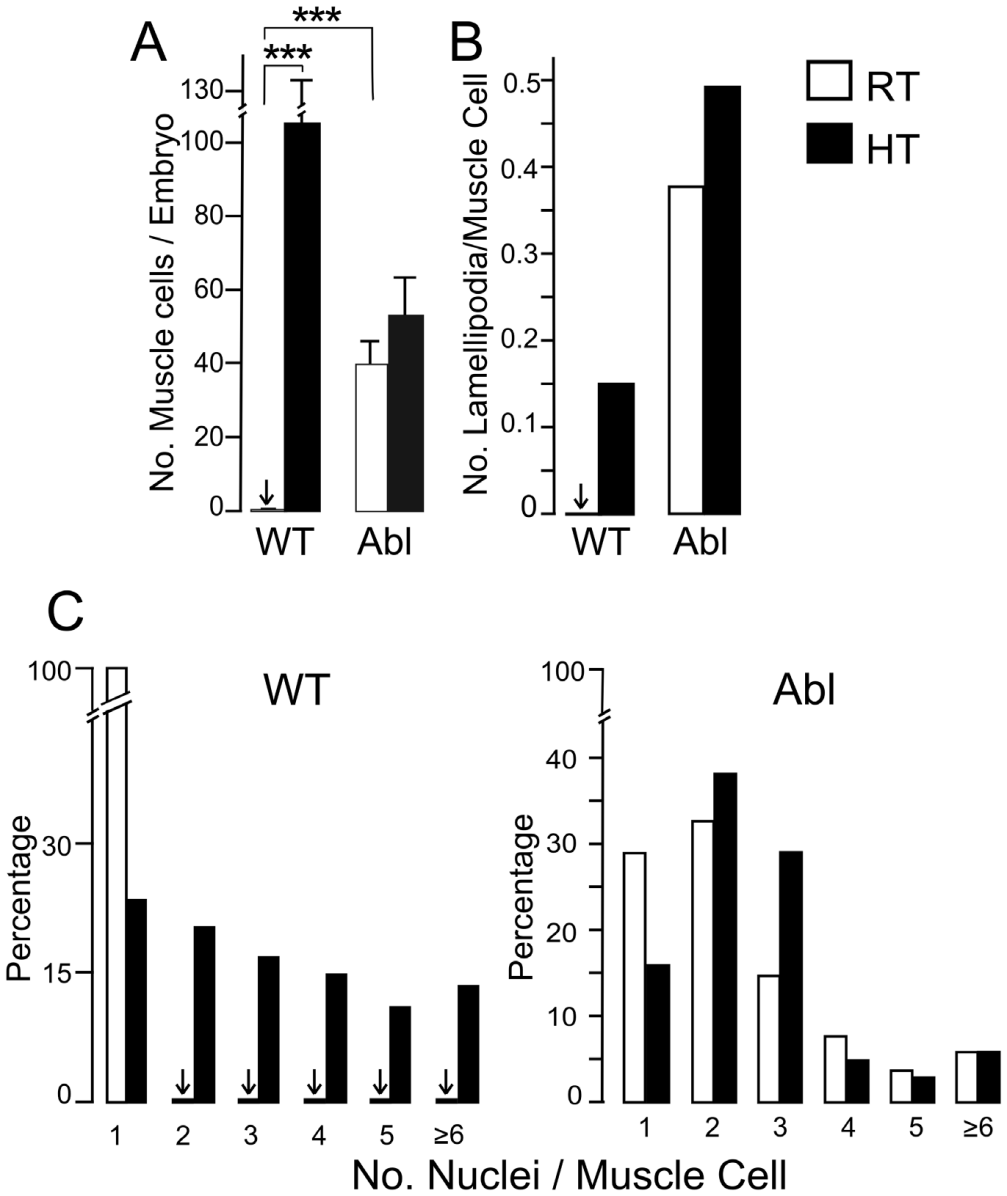
Analysis of WT and *Abl* muscle growth at RT and HT. A) Muscle cell numbers per single-embryo culture from stage 10 WT and *Abl* embryos incubated at RT and HT. At RT, abundant muscle cells were present in *Abl* cultures but nearly absent in WT cultures (***p<0.001, rank test). HT-incubation greatly increased the number of muscle cells in WT cultures (***p<0.0001, rank test) while exerted little effect on *Abl* cultures. Similar proportions between muscle cells isolated from cell clusters and those still associated with clusters were observed among HT WT cultures, RT *Abl* cultures, and HT *Abl* cultures (57%, 41%, and 57% isolated muscle cells, respectively, for the 3 cultures). Error bars, SEM. Sample sizes for A (number of coverslips (C) and number of muscle cells (N) for each culture): WT RT, C = 17, N = 20; WT HT, C = 6, N = 633; *Abl* RT, C = 6, N = 238; *Abl* HT, C = 7, N = 371. Note that muscles cells in WT RT cultures were nearly absent (arrows). B) Average number of lamellipodia per muscle cell. The number for *Abl* RT was greater than both WT RT and WT HT. C) Increased degree of muscle fusion by HT incubation and *Abl* mutations. A majority of muscle cells in each culture type were multinucleated, except for WT RT cultures, in which very few muscle cells were encountered (total 20 in 17 cultures) and they were exclusively mononucleated (arrows). The fusion rate is significantly increased after HT incubation (WT, p<0.0001; *Abl,* p<0.03, χ^2^ test). The identical cell samples from the same cultures were analyzed for B & C (number of muscle cells (N) and number of lamellipodia (n): WT RT, N = 20, n = 0; WT HT, N = 296, n = 39; *Abl* RT, N = 228, n = 60; *Abl* HT, N = 92, n = 44). Same cultures shown in A were analyzed. All cultures were derived from stage 10 embryos and incubated 2–5 days.

We discovered an unexpected, striking effect of HT incubation on myocyte migration and cell fusion in cultures derived from stage 10 embryos (Figs. 6 & 7). This is reminiscent of the normal developmental progression of WT embryos later at stage 12 or older, which initiates myocyte migration and myotube formation (Figs. 1, 2&6).

**Figure 8:**
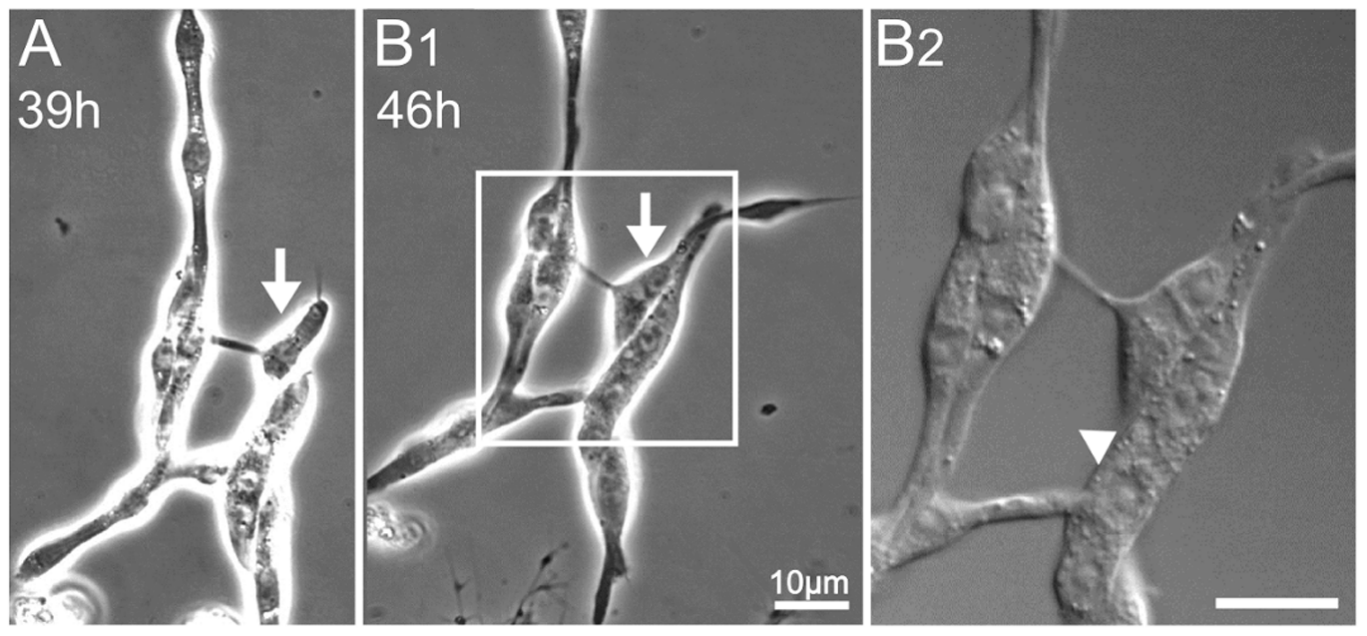
Sample of muscle cell elongation and fusion in HT-incubated WT culture. Time lapse phase contrast images (100X) taken 39 hours (A) and 46 hours (B1) after plating. Arrows denote an example of muscle elongation. B2) Enlarged DIC image of the boxed area in B1. Arrowhead indicates a potential fusion site. All cultures were derived from stage 10 embryos. Scale bars, 10 µm.

Within 4 days in WT stage 10 cultures grown at HT, a higher percentage of muscle cells were fully separated from nerve clusters (Figs. 6 & 7) and many fused myotubes formed with nuclear numbers beyond 7 (Fig. 7A, C). HT muscle cells did display some well-expanded lamellipodia occasionally (Fig. 7B) and many putative innervations or interactions with neurites or growth cones from nerve cells (Fig. 6B2–B3). We observed muscle cells in the process of elongation and fusion at HT, as shown in the time-lapse photography (Fig. 8), mirroring the *in vivo* muscle developmental sequence [27].

It should be noted that in our study these stage 10 embryos followed a normal developmental course at RT up to the time of plating. Therefore, the intrinsic capability of myocyte migration has developed by stage 10, even though the myocytes do not emerge from cell clusters when grown at RT. In support of this notion, stage 8 cultures also displayed some isolated muscles cells or aggregates when incubated at 30°C, although these muscle cells were short-lived and could only survive approximately 2 days after plating (data not shown). In addition, we carried out stage 12 cultures of *Abl* at RT and WT at HT, both of which displayed well-developed muscle cells, with enhanced muscle lamellipodia growth and cell fusion (Fig. S2). Together, these results suggest similar enhancements in muscle development by *Abl* mutation and HT treatment.

One interesting question was whether HT incubation and *Abl* mutations have synergistic effects on muscle development. Following the same protocol of HT incubation for WT, we found that muscle cells in *Abl* single-embryo cultures did not appear to be further promoted in muscle phenotypes. There was no significant increase in the numbers of muscle cells and muscle lamellipodia (Fig. 7A–B). However, the myocyte fusion rate appeared to be slightly increased at HT, as indicated by the increase in multinucleated muscle cells (Fig. 7C). The results from stage 10 *Abl* embryonic cultures demonstrate that HT exerted only limited effects on muscle phenotypes despite its extensive enhancement of *Abl* neuronal phenotypes. The apparent distinctions of the HT effects on neuronal and muscle cell development in *Abl* cultures suggest the possibility of a plateau effect of *Abl* mutations on muscle development that could not be further modified with HT incubation.

**Figure 9:**
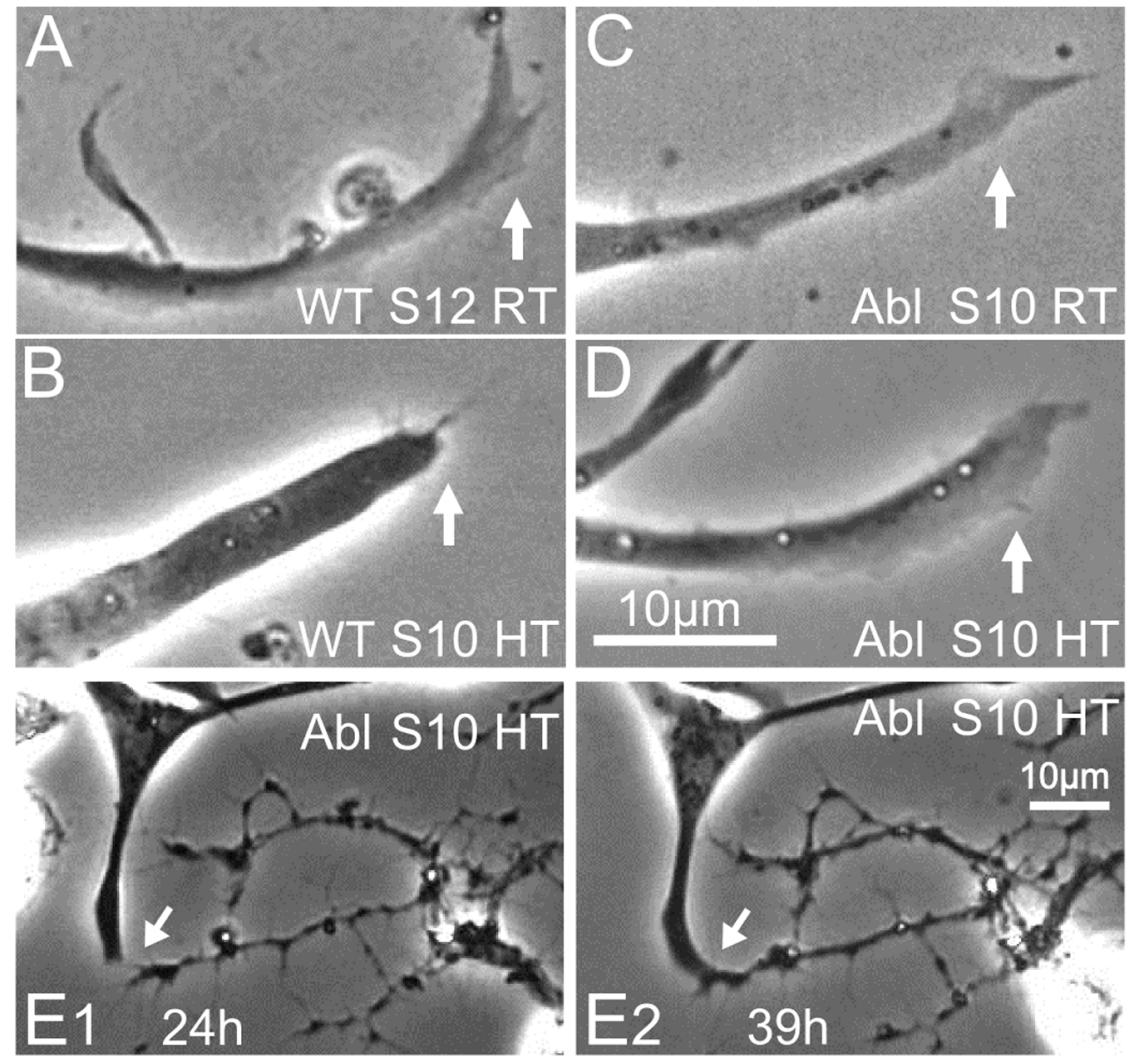
Promotion of muscle lamellipodia development and interaction with neurons in *Abl* cultures. A–D) Samples of phase contrast images (100X) displaying lamellipodia from WT and *Abl* muscle cells in culture. (A) is from cultures incubated 7D, (B–D) are from cultures incubated 24 h–48 h. WT cultures from advanced embryos (stage 12) produce expanded lamellipodia when incubated at RT (A), whereas cultures derived from earlier embryos (stage 10) show muscle cells only after HT incubation, which display microspikes but rarely well extended lamellipodia (B). *Abl* cultures exhibit more abundant lamellipodia at stage 10 following incubation at RT (C) or HT (D). E) Example of neuron-muscle interactions in HT-incubated *Abl* cultures. Time lapse images (phase contrast, 40X) taken 24 hours (E1) and 39 hours (E2) after plating. Neuronal filopodia and muscle lamellipodia first approached each other (E1) and subsequently formed morphological connection (E2). Arrows indicate the interaction site. Scale bars, 10 µm.

Apparently, high-temperature incubation of WT cultures mimics some, but not all, aspects of *Abl* muscle phenotypes. Muscle lamellipodia are a characteristic organelle of cultured embryonic muscle cells, seen often in cultures from more advanced embryos (stage 12), which are thought to play important roles in cell movement and cell-cell interactions. We observed that muscle cells in *Abl* cultures incubated at RT and HT presented far more numerous well-expanded lamellipodia than WT muscle cells grown at HT (Figs. 7B & 9). Furthermore, interacting protrusions from both neuron and muscle in the process of establishing connections were observed using time-lapse photography. This phenomenon was more abundant in *Abl* cultures at both RT and HT (Fig. 9E).

**Figure 10:**
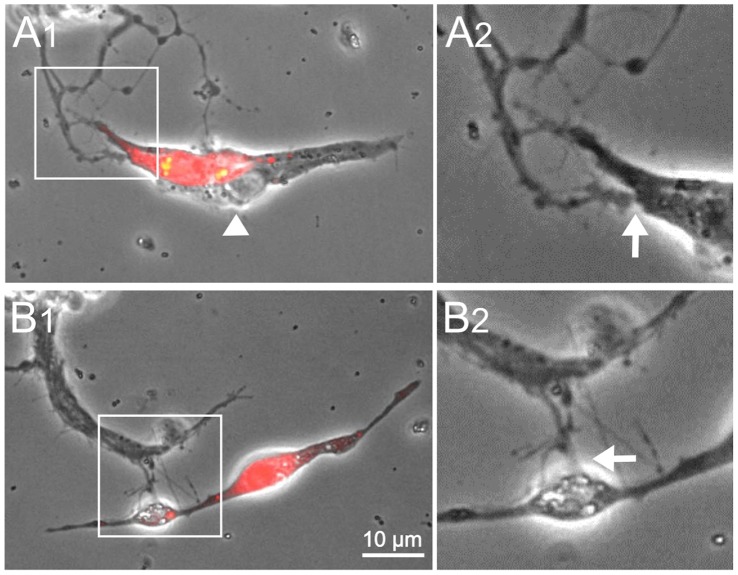
Nerve-muscle interactions between WT and *Abl* cells. A1 & B1) Merged images of phase contrast and fluorescent optics. The cells labeled red are from Da-Gal4>UAS-RFP embryos (*Abl* ^+/+^) and the unlabeled cells are from *Abl* embryos. A1) Neurites from an *Abl* neuronal cluster interact with a group of *Abl* and *Abl* ^+/+^ muscle cells, which are potentially in the process of fusion. A2) Enlargement of the boxed area in A1 showing nerve-muscle contact. B1) *Abl* neurites contact a multinucleated muscle cell, which is labeled by RFP. B2) Enlargement of boxed area in B1. This suggests that *Abl* neurons can make contact with WT muscle cells (and vice versa, data not shown). Age of culture, 3 days. Cultures were derived from stage 10 *Abl* mutants and stage 12 WT embryos. Scale bar, 10 µm.

Despite the apparently altered *Abl* muscle development, the basic properties that define the muscle cell identity seem to be preserved in cultured *Abl* myocytes. To examine whether normal WT nerve and muscle cells could interact with their *Abl* counterparts, we took advantage of RFP-labeled embryos to enable vital marking of developing cells of identified genotypes. The expression on RFP in the tissue of WT embryos was controlled using the ubiquitously expressed Da-GAL4 driver, *daughterless.* We mixed cultures derived from WT and *Abl* embryos and found fusions among WT and *Abl* myocytes to form multinucleated muscle cells (Fig. 10A). Furthermore, neurite projections from WT neurons made contacts with *Abl* myocytes in culture (Fig. 10B). The converse was also true (data not shown). This observation demonstrates that *Abl* mutations do not prevent cell-cell recognition, interaction and fusion with the WT counterparts.

## Discussion

### Recapitulation of Embryonic Muscle and Nerve Cell Development and Alterations by *Abl* Mutations in Dissociated Single-embryo Cell Cultures

We discovered that *Abl* mutations profoundly alter the developmental progression and properties of myocytes, including myocyte movement, lamellipodia formation, and fusion process in the single-embryonic culture. Multinucleated muscle cells and neuron-muscle contacts were present in open fields of *Abl* cultures derived from stage 10, while WT embryos only at a later stage (12), but not at stage 10, could produce isolated single or grouped muscle cells in dissociated cultures. Also, similar to our earlier observations on dissociated larval CNS cultures [24], embryonic *Abl* neurons displayed altered morphology with dark nodules along neurites and more abundant filopodia.

During *in vivo* development, neuroblasts and myoblasts are determined before stage10 [28]. The nervous system starts to develop after gastrulation, while muscle differentiation occurs at stage 12 with signs of cell fusions [21]. There are two temporal phases of *in vivo* fusion events. During the first fusion phase (stage 12–13), founder cells (FCs) fuse with fusion competent myoblasts (FCMs) and a 2–3 nucleated precursor cell forms. Subsequently (stage 14–15), more massive fusion events between precursor cells and FCMs occur until the final size of the muscle is reached [29], [30].

Factors affecting the above developmental progressions in the nerve and muscle systems still remain to be fully elucidated. The *Drosophila* primary embryonic culture systems have been contributing to the studies of cellular development under controlled conditions since the 1970s [31], [32], [33], [34]. Cells from different developmental stages of embryos can differentiate into a variety of cell types in culture [35], [36].

It has been reported that such primary culture systems maintain the formation of *in situ*-like neuron lineages [37], [38], [39]. Our previous studies using the *Drosophila* primary culture system of dissociated neurons from the larval CNS and the embryonic “giant” neuron culture derived from division-arrested neuroblasts have also demonstrated lineage-dependent neuronal development, as well as basic neuronal function via electrophysiology and optical imaging [40], [41], [42], [43].

More recently, muscle cell development has been successfully studied in dissociated cultures to observe morphogenesis and to define effectors in the development process using gastrulae stages. Typically, these studies utilize cultures established by mixing and dispersing the contents from large numbers of embryos that have undergone extensive mechanical dissociation or enzyme treatment [22], [23]. However, it should be noted that in our current culture system, muscle cells do not migrate from cell clusters extracted from single embryos prior to stage 12. This enabled us to detect the striking *Abl* phenotype of muscle migration and maturation.

The crucial technical differences were that we extracted contents from individual embryos and extruded them directly into the culture medium onto untreated glass coverslips. This procedure partially preserved the embryonic environments, especially the aspect of cellular aggregation at the specific stages of embryonic development. This was reflected by the distinct appearance of cell clusters and isolated single cells that enabled us to study, at a subcellular resolution, processes of myocyte movement, fusion and interactions with neurons (Figs. 2 & 3).

In a separate set of experiments, we treated stage 10 embryos with mechanical dissociation and dispersed the mixed contents to establish dissociated cell cultures. Indeed, consistent with published results [22], [23], we observed individual muscle cells undergoing migration and fusion, indicating that embryonic myocytes at stage 10 are capable of migration and fusion when isolated in culture (Fig. S3).

Our results also suggest the possibility that developmental progression of muscle cells is preserved in our primary culture (Fig. 2). The serendipitous finding of the striking effect of *Abl* mutations in this culture system provides a stark contrast of its differential effects on nerve and muscle cells (Fig. 3 & 4), raising the probability that Abl may function in neuron and muscle through distinct interacting partners.

### Abl and its Diverse Interaction Partners in Different Cell Types, Cellular Organelles and Subcellular Compartments

The major phenotypic alterations of *Abl* nerve and muscle cells in our cultures include the enhanced growth of muscle lamellipodia and filopodia along neurites (Figs. 3, 4, 5 & 7). There were also indications that *Abl* neuronal filopodia became more numerous at the expense of growth cone lamellipodia expansion, a defect that could be overcome by HT incubation (Figs. 4 & 5). However, HT incubation did not reduce the dark nodules along neurites, indicating that the production of dark nodules and excessive filopodia are derived from different mechanisms (Fig. S4). Apparently, Abl normally functions to exert inhibitory growth control of neuronal filopodia, and possibly muscle lamellipodia as well (Figs. 4 & 9).

In *Drosophila* and other species, Abelson tyrosine kinase has been implicated in cell migration, adhesion, cell-cell interaction, and morphogenesis in a variety of tissues during development [8], [11], [44], [45], [46]. Among these functional and developmental regulations, many of the Abl-regulated neuronal and muscular processes are related to cytoskeleton dynamics [5], [47]. It is known that Abl family kinases convey signals from cell surface receptors for growth factors, such as platelet derived growth factor (PDGF), epithelial growth factor (EGF), netrin, and adhesion receptors, such as cadherin, to regulate cytoskeletal dynamics by phosphorylating several regulatory proteins, such as β-catenin [45], [48], [49].

For example, during axonogenesis, in *Drosophila*, Abl regulates cell adhesion and actin polymerization through suppressive/antagonistic interactions with its substrates, the Ena/VASP family of actin regulatory proteins [10], [50] and Abl interacting protein, Abi [51]. Moreover, Ena functions together with Diaphanous (Dia)-related formins, to regulate the balance between filopodia and lamellipodia. It is known that the presence of either Dia or Ena, is sufficient to induce filopodia growth, but only together will they induce lamellipodia protrusion. Interestingly, constitutively active Dia increased filopodia growth, mimicking our *Abl* neuronal phenotype [52]. It will be interesting to investigate in future studies whether our observations of filopodia overgrowth in *Abl* neurons also involves dysregulation of Dia-related formins. In vertebrates, c-Abl and Ena/VASP also regulate the leading edge mediated by a specific intermediary protein, Lamellipodin, which is required for lamellipodia formation during axonal morphogenesis [53], [54]. It will be important to identify the *Drosophila* homologous gene(s) encoding Lamellipodin-related protein(s) in order to elucidate the mechanism of enhancing muscle lamellipodia formation and its relation to muscle cell movement and fusion, and to explain the apparently opposite effects on neuronal growth.

In addition to the identification of Abl interacting partners, another important issue is the temperature dependence of these protein activities. Such information may provide insights into our findings, specifically that HT incubation promotes myocyte migration out of cell clusters (Figs. 6 & 7) mimicking the *Abl* muscle phenotype, enhances neuronal lamellipodial growth, and reverts *Abl* neuronal filopodium-lamellipodium phenotypes.

**Figure 11:**
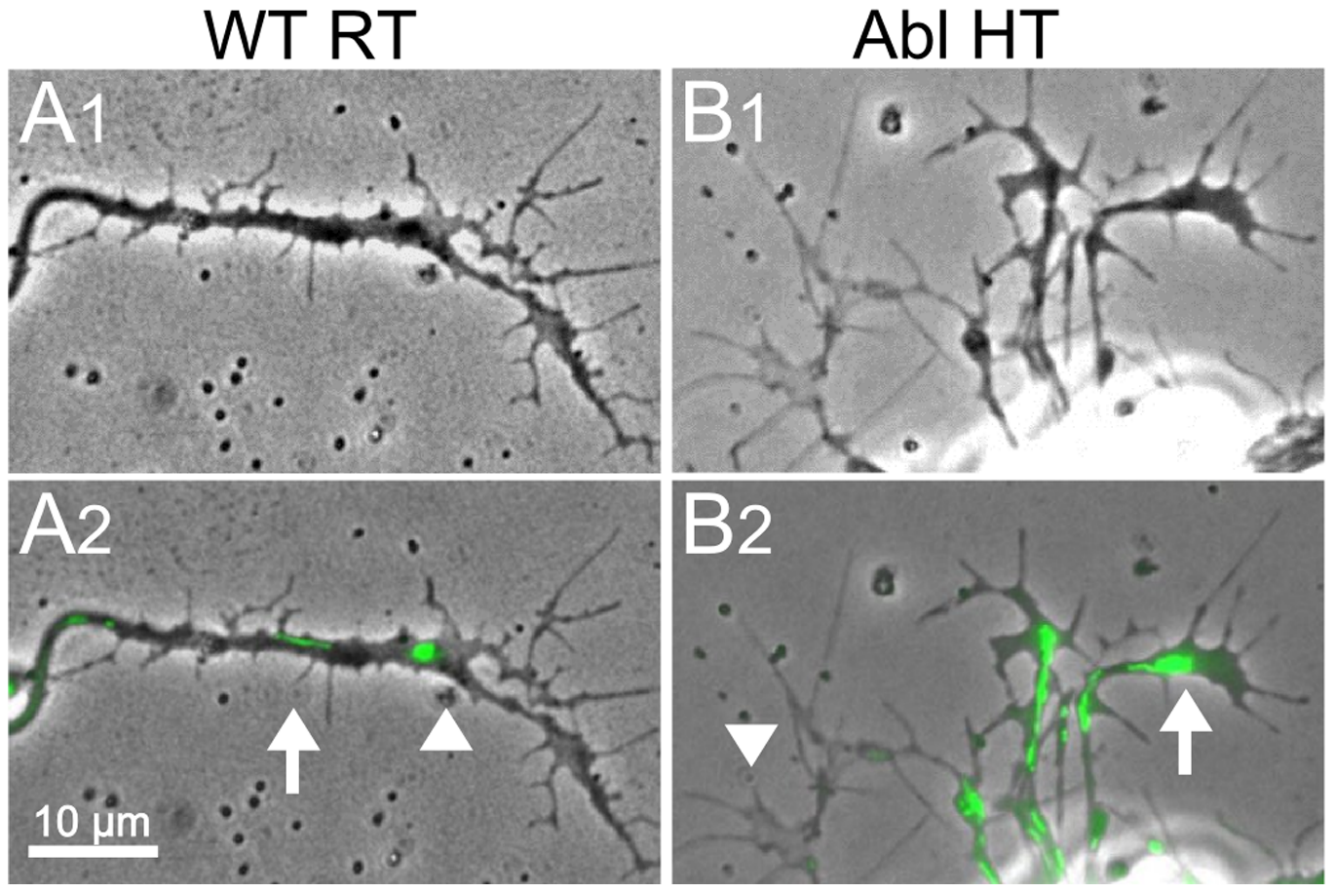
Mitochondria are highly enriched in the dark nodules along neurites of *Abl* neurons. A1–B1) Phase contrast images of phase dark nodules and phase light lamellipodia from WT (RT) and *Abl* (HT) neutires (100X). A2–B2) Merged fluorescent and phase contrast images showing locations of Rh123 staining. Arrowheads, phase light growth cones. Arrows, phase dark structures along the neurite that accumulate Rh123 staining. The staining indicates that dark nodules in *Abl* are enriched with mitochondria and possibly other organelles. Age of cultures, 2–4 days. All cultures were derived from stage 10 embryos. Scale bar, 10 µm.

In the current study, we have focused on the alterations of the cellular organelles lamellipodia and filopodia in *Abl* cultures. However, in *Abl* neurons, another striking phenotype is the characteristic dark nodules along the neurites and darkened central domains of the growth cone (Fig. 4) [24]. Our initial efforts indicated that these structures may be enriched in membranous organelles, such as mitochondria, as evidenced by Rhodamine 123 staining (Fig. 11). In phalloidin staining experiments, these regions are also enriched with actin, suggesting abnormal actin filament organization (data not shown). It should be noted that dark nodules in *Abl* neurons grown at HT were still abundant and enriched with organelles such as mitochondria (Fig. S4), a further contrast with the HT mitigation of filopodial defects. Future experiments should determine whether additional cellular organelles accumulate in these structures. This may provide indications for modifications in trafficking of these organelles, which is another important cellular mechanism dependent upon functional cytoskeletal cables.

In future studies, it will be important to determine the differential partners of Abl in the regulation of lamellipodia and filopodia in neuron and muscle cells and their temperature-dependent properties. Our culture system offers the potential of growing nerve and muscle cells with identified lineages and with vital cell markers, thus enabling the study of interactions among nerve and muscle cells of different genotypes which can then be monitored at high temporal and spatial resolutions.

## Supporting Information

Figure S1 **Consistent neuron and muscle phenotypes observed in** ***Abl*** **^4^/+ and** ***Abl^1^/Abl^1^*** **from dissociated stage 10 embryos.** Phase contrast images showing similar *Abl* mutant phenotypes observed in cultures from stage 10 embryos of different lines (100X). For genetic background control, the heterozygous *Abl* ^4^ stock used for the majority of the experiments in this study (kept over a third chromosome balancer, see Methods) was out crossed to WT (CS) to obtain a new line of *Abl* ^4^ heterozygotes (denoted below *Abl* ^4^/+). WT cultures show abundant neuronal clusters but rarely muscle cells (A) while both neurons and muscle cells are abundant in *Abl* ^4^/+ (B) and *Abl* ^1^/*Abl* ^1^ (C) cultures. Arrows point to dark nodules (B1 and C1) and phase light growth cone (A). B2–C2) Neurons making contact with multinucleated muscle cells in both *Abl* ^4^/+ and *Abl* ^1^ cultures. Arrows, nerve-muscle contacts; arrowheads, muscle nuclei. C3)Well-expanded lamellipodia (arrow) of a muscle cell in *Abl^1^* culture. Age of cultures, 2–4 days. Scale bar, 10 µm.
(TIF)

Figure S2 **Movement and fusion of muscle cells observed in WT,** ***Abl*** **, and WT HT-incubated cultures dissociated from stage 12 embryos.** Sample DIC images of cultures derived from stage 12 embryos (40X). A) WT cultures incubated at room temperature, B) *Abl* cultures incubated at room temperature, C) WT cultures incubated at HT. Age of cultures, approximately 2 days. All displayed muscle cells, both isolated (arrows) and associated with cell clusters (arrowheads). (D1–D2) sample of muscle lamellipodia (arrows) from stage 12 WT RT and HT cultures, 100X. E) Portion of muscle cells displaying lamellipodia increased after HT incubation in cultures derived from stage 12. One pair of RT and HT cultures incubated for about 24 h was used for this statistics. RT, muscle numbers, N = 83, lamellipodia number, n = 13; HT, N = 69, n = 32. Scale bar, 20 µm.
(TIF)

Figure S3 **Phase contrast images comparing cultures from embryos treated with different dissociation methods.** A) Muscle cells are rarely seen in the single-embryo culture used in the current study. B1) Multinucleated muscle cells (arrows) are abundant in mechanically dissociated cultures (several embryos after homogenization, see Methods). B2–B3) Time lapse images showing morphological changes of muscle lamellipodia (arrows) from 18 to 24 hours after plating. 20X. All cultures were derived from stage 10 embryos. Scale bars, 20 µm.
(TIF)

Figure S4 **Dark nodules along neurites kept in both RT and HT cultures.** Phase contrast images from *Abl* cultures derived from stage 10 (100X). A) *Abl*, RT, B) *Abl*, HT. Dark nodules displayed in both RT and HT cultures (arrowheads), however enlarged lamellipodia were readily observed in HT cultures (arrow). Age of cultures, 3–4 days. Scale bar, 10 µm.
(TIF)
