# Supplementary figures and images for: Distinct Effects of Abelson Kinase Mutations on Myocytes and Neurons in Dissociated Drosophila Embryonic Cultures: Mimicking of High Temperature
Source: PLoS One. 2014 Jan 21;9(1):e86438. doi: 10.1371/journal.pone.0086438 (PMC3897706; doi:10.1371/journal.pone.0086438)

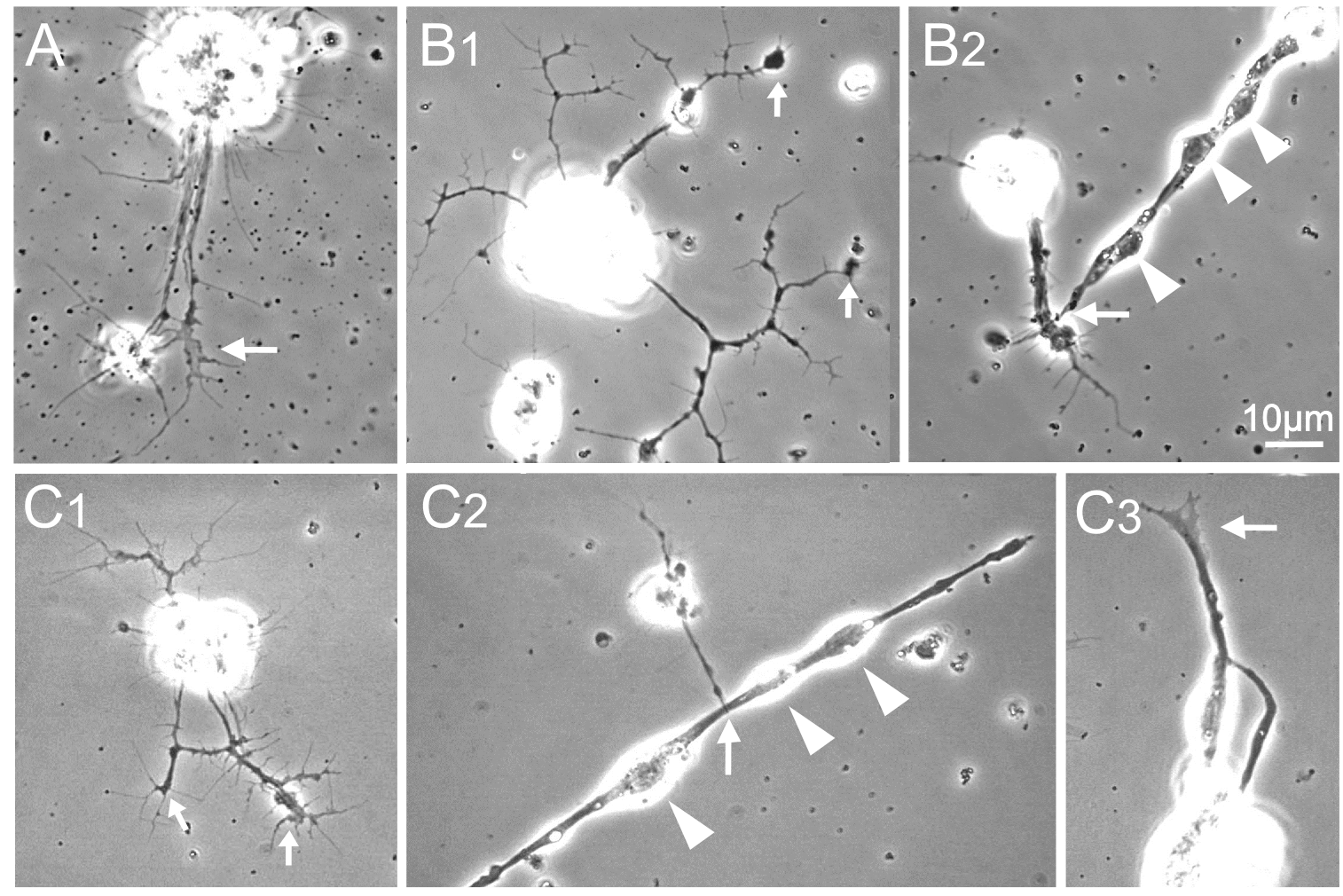

Supplement: Figure S1 — Consistent neuron and muscle phenotypes observed in Abl 4/+ and Abl1/Abl1 from dissociated stage 10 embryos. Phase contrast images showing similar Abl mutant phenotypes observed in cultures from stage 10 embryos of different lines (100X). For genetic background control, the heterozygous Abl 4 stock used for the majority of the experiments in this study (kept over a third chromosome balancer, see Methods) was out crossed to WT (CS) to obtain a new line of Abl 4 heterozygotes (denoted below Abl 4/+). WT cultures show abundant neuronal clusters but rarely muscle cells (A) while both neurons and muscle cells are abundant in Abl 4/+ (B) and Abl 1/Abl 1 (C) cultures. Arrows point to dark nodules (B1 and C1) and phase light growth cone (A). B2–C2) Neurons making contact with multinucleated muscle cells in both Abl 4/+ and Abl 1 cultures. Arrows, nerve-muscle contacts; arrowheads, muscle nuclei. C3)Well-expanded lamellipodia (arrow) of a muscle cell in Abl1 culture. Age of cultures, 2–4 days. Scale bar, 10 µm. (TIF) [file pone.0086438.s001.tif]

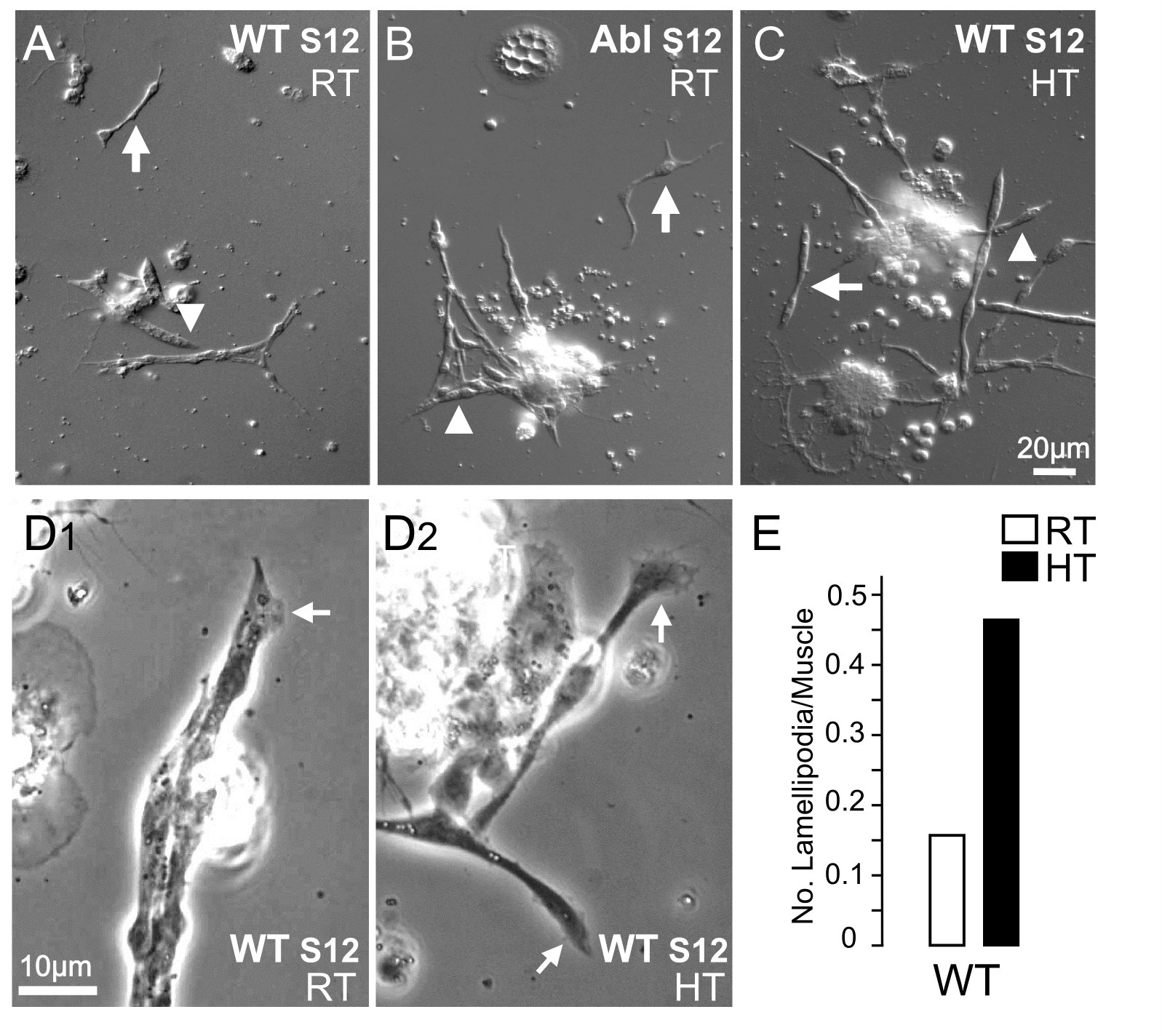

Supplement: Figure S2 — Movement and fusion of muscle cells observed in WT, Abl , and WT HT-incubated cultures dissociated from stage 12 embryos. Sample DIC images of cultures derived from stage 12 embryos (40X). A) WT cultures incubated at room temperature, B) Abl cultures incubated at room temperature, C) WT cultures incubated at HT. Age of cultures, approximately 2 days. All displayed muscle cells, both isolated (arrows) and associated with cell clusters (arrowheads). (D1–D2) sample of muscle lamellipodia (arrows) from stage 12 WT RT and HT cultures, 100X. E) Portion of muscle cells displaying lamellipodia increased after HT incubation in cultures derived from stage 12. One pair of RT and HT cultures incubated for about 24 h was used for this statistics. RT, muscle numbers, N = 83, lamellipodia number, n = 13; HT, N = 69, n = 32. Scale bar, 20 µm. (TIF) [file pone.0086438.s002.tif]

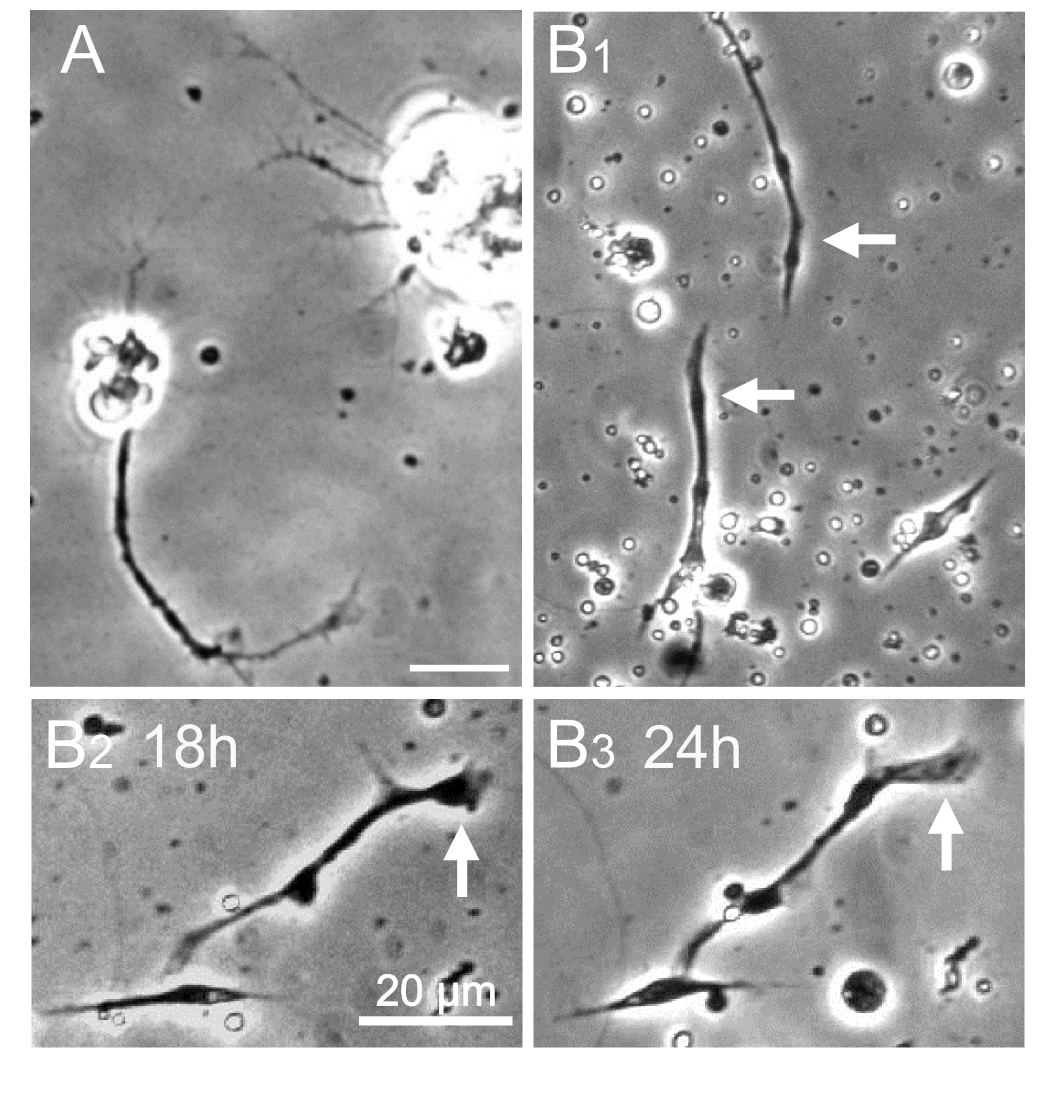

Supplement: Figure S3 — Phase contrast images comparing cultures from embryos treated with different dissociation methods. A) Muscle cells are rarely seen in the single-embryo culture used in the current study. B1) Multinucleated muscle cells (arrows) are abundant in mechanically dissociated cultures (several embryos after homogenization, see Methods). B2–B3) Time lapse images showing morphological changes of muscle lamellipodia (arrows) from 18 to 24 hours after plating. 20X. All cultures were derived from stage 10 embryos. Scale bars, 20 µm. (TIF) [file pone.0086438.s003.tif]

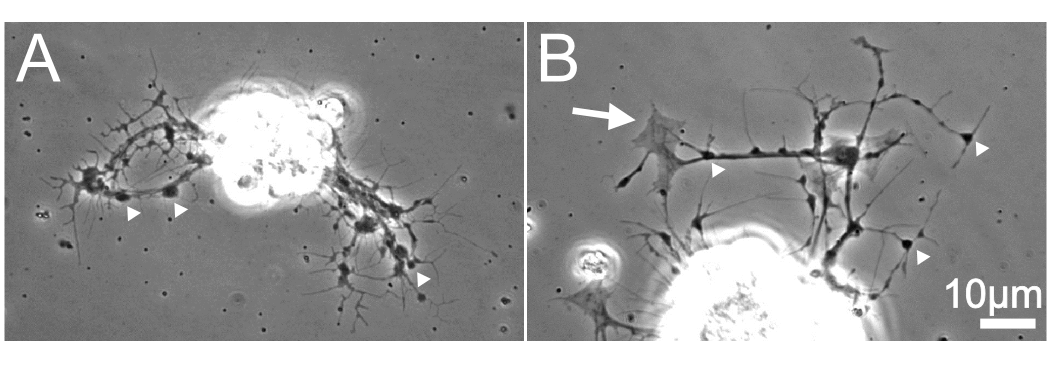

Supplement: Figure S4 — Dark nodules along neurites kept in both RT and HT cultures. Phase contrast images from Abl cultures derived from stage 10 (100X). A) Abl, RT, B) Abl, HT. Dark nodules displayed in both RT and HT cultures (arrowheads), however enlarged lamellipodia were readily observed in HT cultures (arrow). Age of cultures, 3–4 days. Scale bar, 10 µm. (TIF) [file pone.0086438.s004.tif]
